# Challenging interpretation of low-level *PTCH1* mosaicism in patients with clinically diagnosed Gorlin syndrome: a case series and review of the literature

**DOI:** 10.1186/s13053-026-00332-3

**Published:** 2026-03-26

**Authors:** Tanya M. Dwarte, Rozanna Alli, Shweta Srinivasa, Fallon Noon, Sumudu Perera Kimmantudawage, Lisa Gordon, Victoria Beshay, Anthony M. Joshua, Raquel Ruiz Araujo, Chris Jalilian, David M. Thomas, Miriam J. Smith, Ingrid Winship, Mandy L. Ballinger, Minmin Li, Katherine M. Tucker, Eliza K. Courtney

**Affiliations:** 1 Hereditary Cancer Centre, Prince of Wales Hospital, Randwick, NSW Australia; 2 Genetic Health Queensland, Brisbane, QLD Australia; 3 Family Cancer Service, Westmead Hospital, Westmead, NSW Australia; 4 Genomic Medicine, Royal Melbourne Hospital, Parkville, VIC Australia; 5 Molecular Pathology, Peter MacCallum Cancer Centre, Melbourne, VIC Australia; 6 The Kinghorn Cancer Centre, St Vincent’s Hospital, Garvan Institute of Medical Research, Darlinghurst, NSW Australia; 7 School of Clinical Medicine, UNSW Medicine and Health, UNSW Sydney, Sydney, NSW Australia; 8 Department of Dermatology, Westmead Hospital, Westmead, NSW Australia; 9 Sydney Medical School, Faculty of Medicine and Health, The University of Sydney, Sydney, NSW Australia; 10 Skin Health Institute, Melbourne, VIC Australia; 11 Monash Health, Melbourne, VIC Australia; 12 Centre for Molecular Oncology, UNSW Sydney, Sydney, NSW Australia; 13 Omico, Sydney, NSW Australia; 14 Division of Evolution, Infection and Genomics, School of Biological Sciences, Faculty of Biology, Medicine and Health, University of Manchester, Manchester Academic Health Science Centre, Manchester, UK; 15 Manchester Centre for Genomic Medicine, St Mary’s Hospital, Central Manchester University Hospitals, NSH Foundation Trust, Manchester Academic Health Science Centre, Manchester, UK; 16 Department of Medicine, University of Melbourne, Parkville, VIC Australia; 17 Children’s Cancer Institute, Lowy Cancer Centre, UNSW Sydney, Sydney, NSW Australia; 18 Kids Cancer Centre, Sydney Children’s Hospital, High Street, Randwick, NSW Australia

**Keywords:** Gorlin syndrome, Mosaicism, Nevoid Basal Cell Carcinoma syndrome, PTCH1, Segmental

## Abstract

**Background:**

Individuals meeting clinical diagnostic criteria for Gorlin syndrome typically harbor germline pathogenic variants in *PTCH1* or *SUFU*. The molecular basis of a proportion of cases remains unresolved, with mosaicism a possible explanation for some of these unexplained cases.

**Case presentations:**

We describe four probands meeting clinical diagnostic criteria for Gorlin syndrome with confirmed mosaic pathogenic variants. The first proband, a 38-year-old male with metastatic basal cell carcinoma and over 30 prior lesions, had no reportable variants identified on peripheral blood DNA. Somatic analysis of a metastatic lymph node identified a pathogenic *PTCH1* variant, subsequently detected at low levels (< 3% variant allele frequency (VAF)) in multiple non-tumor tissues, consistent with mosaicism. The second proband, a 39-year-old female with a history of > 250 basal cell carcinomas, had previously undergone *PTCH1* and *SUFU* testing on peripheral blood DNA, with no reportable variants identified. Somatic testing arranged on three basal cell carcinomas identified a common *PTCH1* frameshift variant, which on manual re-analysis of peripheral blood DNA was present at < < 1% VAF. Two additional probands, a 32-year-old female and a 30-year-old male, had a *PTCH1* variant reported at 11% and 15% VAF, respectively, in peripheral blood DNA. The clinical features of these individuals are described and compared with 11 additional mosaic probands with confirmed *PTCH1* variants and/or clinically diagnosed Gorlin syndrome identified from the literature.

**Conclusions:**

The presented case series and accompanying literature review provide the largest summary to date of the spectrum of clinical features observed due to *PTCH1* mosaicism. Importantly, it highlights the possibility that low-level *PTCH1* mosaicism may still result in a classic Gorlin syndrome phenotype. This review reinforces the importance of additional testing of multiple tissue types to achieve a molecular diagnosis, given the implications for familial risk assessment, reproductive planning, and to support access to targeted systemic therapies.

**Supplementary Information:**

The online version contains supplementary material available at 10.1186/s13053-026-00332-3.

## Introduction

Gorlin syndrome (GS), also known as Gorlin-Goltz syndrome (GGS) and Nevoid Basal Cell Carcinoma syndrome (NBCCS), is a rare, autosomal dominant condition predominantly caused by germline pathogenic (or likely pathogenic) variants (PV/LPV) in *PTCH1* [OMIM #109400] and *SUFU* [OMIM #620343] [1]. Scarce reports of *PTCH2* variants in GS [2] remain controversial with more recent studies demonstrating these are unlikely to be causative [3, 4]. GS is characterized by multiple (> five) or young-onset basal cell carcinoma(s) (BCCs), with additional benign and malignant lesions, phenotypic and developmental abnormalities (Table 1) [5]. Whilst there is no formal consensus on clinical diagnostic criteria, it is generally accepted that a clinical diagnosis of GS is established when either two major criteria and one minor; or one major and three minor criteria are present [5, 6]. Whilst highly penetrant, phenotypic variability is common and 20–30% of cases arise from a *de novo PTCH1* PV [7].

**Table 1:** Gorlin syndrome clinical diagnostic criteria and literature review of mosaic clinically diagnosed and *PTCH1* variant presentations

Reference	Summary of clinical features in reported mosaic clinically diagnosed and PTCH1 variant cases														
	Current study				Shelley et al.[8]	Camisa et al. [9]	Gutierrez and Mora [10]	Kansal et al. [11]	Reinders et al. [12]	Reinders et al. [13]		Saeidian et al. [14]	Katayama et al. [15]	Verkouteren et al. [16]	Roemen et al. [17]
Proband characteristics	**Proband 1** 38yo, M	**Proband 2** 39yo, F	**Proband 3** 32yo, F	**Proband 4** 30yo, M	43yo, M	42yo, F	46yo, M	Reproductive age, M (7 mo proband’s father)	22yo, F	41yo, F	36yo, F	31yo F	19yo, M	Mid-40s, F	68yo, M (proband’s father)
Molecular or clinical diagnosis	*PTCH1* NM_000264.3 Exon 9 c.1228_1229del p.(Ser410Cysfs*26)	*PTCH1* NM_000264.5 Exon 2 c.258_259del p.(Leu87Ilefs*2)	*PTCH1* NM_000264.3 Exon 8 c.1093 C > T p.(Gln365Ter)	*PTCH1* NM_000264.3 Intron 18 c.3169–2 A > G p.(Val1057_Leu1102del)	Clinical	Clinical	Clinical	*PTCH1* NM_000264.3 Exon 14 c.2391 C > A p.(Tyr797Ter)	*PTCH1* NM_000264.3 Exon 13 c.1810G > T p.(Glu604Ter)	*PTCH1* NM_000264.4 Exon 14 c.2197_2198del p.(Ser733Ilefs*4)	*PTCH1* NM_000264.4 Exon 15 c.2460 C > G p.(Tyr820Ter)	*PTCH1* NM_000264.3 Exon 15 c.2336-2337insGGTAGGA p.(Asp779Glufs*13)	*PTCH1* NM_000264.4 Exon 10 c.1350delC p.(Leu450fs)	*PTCH1* NM_000264.4 Exon 15 c.2359G > T p. (Glu787Ter)	*PTCH1* NM_000264.5 c.(394+1_395-1)_(1728+1_1729-1)dup Duplication of Exons 3–12
Mutation burden	See Table 2 for full summary	3 BCC 4 reads PB	11% VAF PB	15% VAF PB	NA	NA	NA	VAF not specified, but suggestive of mosaicism	13.5% PB; 17% buccal mucosa; 17.3% UDEC 36.1% tumor	4 BCC; 0–9% VAF NLS; Absent from PB Second hit detected	2 BCC; 1% VAF PB Second hit detected	2 BCC; Absent from PB/NLS	VAF 32.7% BCC 11.6% oral mucosa 9.8% nail 13.8% NLS	2 BCC Meningioma Absent from PB LOH detected	CNV gain ~23% PB; 74% hair; 59% saliva
Segmental effect	No	Yes – more severe on right side	No	No	Yes - quadrant	Yes - unilateral	Yes - unilateral	No	No	Yes - unilateral	No	Yes - Blaschkoid distribution	No	No	No
Clinical Dx criteria fulfilled [6]	Yes	Yes	Yes	Yes	No	No	Yes	Not confirmed	No	No	Yes	No	Yes	No	No
**Major Criteria**															
Lamellar calcification of the falx or clear evidence of calcification < 20yo	(+)	(+)	+	(+)		(+)	+	+							
Odontogenic keratocyst		+ - unilateral	+	+	+ - unilateral						+		+ - single	+	
≥ 2 palmar/ plantar pits		+	+	+		+	+		+		+				
Multiple or young-onset BCCs (Dx < 20yo or > 5 in a lifetime)	+ (> 30 from 21yo)	+ (> 250 from 19yo)	+ (~ 50 from teenage years)	+ (~ 50 from 15yo)	+ (> 50 from childhood)	+ (~ 50 from 12yo)	+ (> 50 from 16yo)		+ (3 lesions)	+ (100s of BCCs removed)	+ (several from 27yo)	+ (multiple superficial lesions from 15yo)	+ (1, 19yo)	+	+ (multiple at older age)
Childhood medulloblastoma													+ (aged 1yo)		
First degree relative with Gorlin Syndrome								(+) – suspected in 7mo son							+
**Minor Criteria**															
Lympho-mesenteric or pleural cysts															
Macrocephaly (OFC > 97th percentile)	+	+	+	(+)^				+							
Cleft lip/palate															
Vertebral/rib anomalies (e.g. bifid/splayed/ extra ribs; bifid vertebrae; scoliosis)		(+)	NA				+	(+) - fusion of some costal cartilages			+				
Preaxial or post axial polydactyly															
Ovarian/cardiac fibromas															
Ocular anomalies (e.g. cataract; developmental defects; hypertelorism; pigmentary changes of the retinal epithelium)	+		NA				+								
Further comments															
Other reported clinical features	Frontal bossing Hypothyroidism	Hashimoto’s thyroiditis	Hyperthyroidisms		Thyroid nodule (38yo)		Frontal bossing Short distal phalanx of both thumbs	Dysmorphic features (not otherwise specified) Enlarged ventricles		Also has *BRCA2* PV Breast cancer			Osteosarcoma (right tibia, 8yo) Therapy-related MDS (11yo)	Meningioma (mid 40s)	

+ Feature present in proband; (+) Feature suspected in proband but not confirmed (e.g. not lamella calcification; unknown if present at age specified; incomplete information of previous clinical investigations etc.); ^ OFC self-reported measurement and not reassessed due to COVID-19 restrictions preventing in-person consultations at the time of genetics review; NA Feature not assessed

Abbreviations: BCC, basal cell carcinoma; BCN, basal cell naevi; CNV, copy number variant; F, female; LOH, loss of heterozygosity; M, male; mo, months old; NLS, non-lesional skin; OFC, occipitofrontal circumference; PB, peripheral blood DNA; PV, pathogenic variant; UDEC, urine derived endothelial cells; VAF, variant allele frequency; yo, years old

Several articles were excluded from this review as listed below. Wicking et al. [18]. reported a family with potential *PTCH1* gonadal mosaicism based on chromosome 9q linkage analysis and there was subsequent detection of a 76-bp deletion in the affected family members [19]. Similarly, Klein et al. [20] found a 21-base-pair-deletion in Exon 9 of *PTCH1* in two affected siblings which was not observed in the peripheral blood DNA of either parent. These studies were excluded as neither parent suspected of gonadal mosaicism showed any features of GS. Musani et al. [21]. reported monozygotic twins with a *de novo PTCH1* c.3364_3365delAT PV, whose father had multiple jaw cysts diagnosed several years before. It was not possible to determine the strand of origin of the *PTCH1* PV. As the twin’s paternal grandfather also had jaw cysts in later life, gonadal mosaicism in the father could not be confirmed. Torrelo et al. [22] was excluded as the patient had paternally-inherited GS with segmental mosaicism of a second *de novo PTCH1* PV conferring increased disease severity. Similarly, Ikemoto et al. [23]. was excluded as the proband had a *de novo* germline *PTCH1* PV and a second somatic *PTCH1* mutation was observed due to suspected reversion error. Igaz et al. [24]. report a 23yo proband with a heterozygous *PTCH1* PV and a second somatic *PTCH1* 5-necleotide deletion PV at lower VAF in bilateral PEComas. They were unable to determine if the two *PTCH1* variants were in cis or trans. Abi Karam et al. [25] reported an individual with two BCCs containing the same *PTCH1* variant suggesting the possibility of GS mosaicism, but this case was excluded as no clinical assessment of this individual was possible. Aradhya et al. [26]. detected mosaic *PTCH1* deletions in two clinically affected individuals using exon-level comparative genomic hybridization (CGH) array (and confirmed via MLPA, though the level of mosaicism was not determined); however, their specific clinical features were not reported. Soufir et al. [27]. report five patients with *typical* GS in which no *PTCH1* variant was identified. Whilst they speculate mosaicism as a potential explanation, these cases were excluded as the clinical features of these individuals were not reported, and a mosaic cause was not confirmed. Zhang et al. [28] reported a 4yo girl diagnosed with a sporadic cardiac fibroma, in which somatic copy number losses encompassing *PTCH1* were identified. This proband was excluded as broader clinical assessment for GS was not included, and we interpreted the variant as most likely representing a tumour-restricted somatic variant rather than mosaicism affecting additional tissues. Finally, two additional studies reporting Gorlin syndrome probands harboring somatic *SMO* variants [29, 30] were excluded as more recent publications have countered these probands have an overlapping condition, termed mosaic *SMO* syndrome

In approximately 25% of GS cases, the underlying molecular basis remains unresolved [3]. Current Next-Generation Sequencing (NGS) technologies may have limited ability to detect certain causative PV/LPVs such as deep intronic variants [31, 32] mobile element insertions [32], or structural variants better detected by alternate methodologies, including multiplex ligation-dependent probe amplification (MLPA) and digital-droplet PCR [17]. Furthermore, RNA analysis of *PTCH1* may be required to identify variants difficult to detect with typical exonic short-read sequencing. It is also hypothesized that as yet unidentified gene(s), likely within the hedgehog signaling pathway, will account for additional unexplained cases [31]. Moreover, somatic mosaicism is increasingly reported in the literature in patients meeting clinical GS diagnostic criteria [8, 10, 12, 13]. It remains challenging to conclusively distinguish all unresolved cases from mosaic presentations due to these limitations in current diagnostic sequencing approaches, which are not validated for low-level sequence variants. Similarly, mosaicism may be tissue-limited and absent (or present at undetectable thresholds) in peripheral blood and skin [33], which are typical DNA sources for routine genetic assessment.

Establishing a molecular diagnosis in individuals with clinically diagnosed GS is critical for guiding clinical management and informing genetic counseling recommendations for at-risk relatives. Here, we present four probands with clinically diagnosed GS, where establishing their molecular diagnosis proved challenging. Additionally, we provide a literature review of previously reported mosaic presentations.

## Case presentation 1

**Fig. 1:**
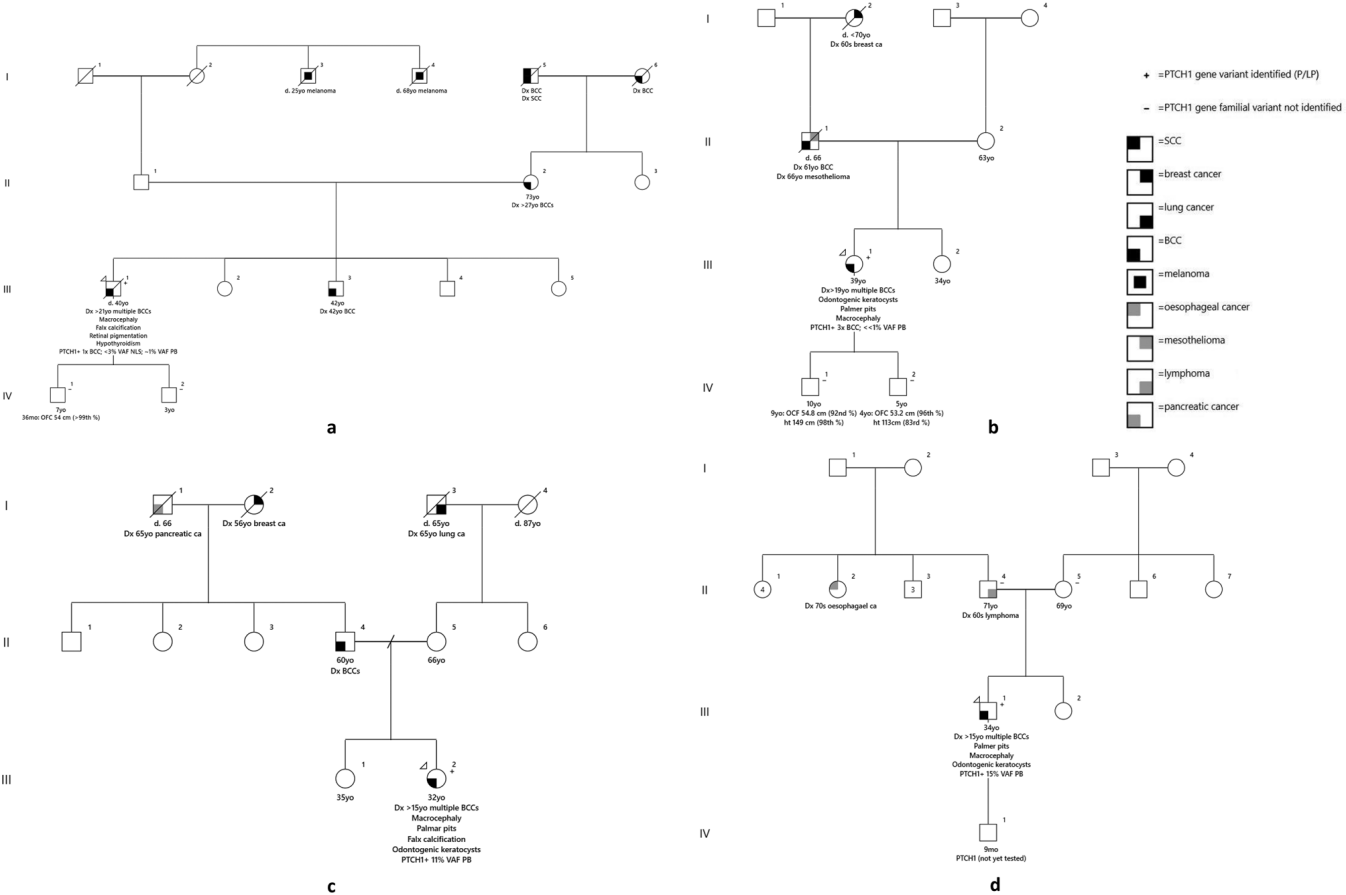
Pedigree of **a** Proband 1 **b** Proband 2 **c** Proband 3 and **d** Proband 4. In each pedigree, the proband is indicated by the arrow. All probands were born to non-consanguineous parents. Whilst some relatives had a history of skin cancers, including BCCs, none of these relatives were reported to have any additional features of GS. Proband 1 had English/Irish ancestry. His mother (II-2), brother (III-4) and both maternal grandparents (I-5 and I-6) were reported to have had at least one BCC. All individuals with skin cancer(s) were described as having pale complexion (Fitzpatrick Type II). Ophthalmological review for IV-1 at age 7 years did not identify pigmentary changes. Neither son had odontogenic keratocysts detected following dental review. Proband 2 had German and English ancestry. Her father (II-1) had two BCCs diagnosed in sun exposed areas after 60 years. Her two sons (IV-1 and IV-2) had borderline macrocephaly but were also of taller stature. Both sons had reportedly normal echocardiograms several years ago, skin examinations were unremarkable and neither had palmar pits. Individual IV-1 had a reported normal chest x-ray and skull x-ray two years prior with a normal dental examination and OPG several months prior to review. Individual IV-2 had recently undergone a normal magnetic resonance imaging (MRI) of the brain for investigation of persistent headaches. Proband 3 had Australian ancestry of white European descent. Her father had a few later-onset BCCs but no other features of GS. Broader family history was not suggestive of inherited cancer predisposition. Proband 4 had Maltese and English ancestry. His mother had multiple benign skin lesions removed, and his father was diagnosed with lymphoma in his 60s. Abbreviations: %, percentile; BCC, basal cell carcinoma; ca., cancer; d., died; Dx, diagnosed; mo, months old; NLS, non-lesional skin; OFC, occipitofrontal circumference; PB, peripheral blood; SCC, squamous cell carcinoma; VAF, variant allele frequency; yo, years old

Proband 1 (III-1 in Fig. 1a) presented at age 38 years with metastatic BCC to his left axilla and extensive bony disease. Since age 21 years, he had been diagnosed with at least 30 BCCs (on both sun-exposed and non-sun-exposed areas bilaterally), despite self-reported minimal sun exposure and diligent sun-safe behaviors. On examination, he had macrocephaly (occipitofrontal circumference (OFC) 60.5 cm, > 99th percentile, + 3.8 SD; height 181 cm, 73rd percentile), hypertelorism, and coarse facial features with frontal bossing. No palmar or plantar pits were identified following soaking in warm water for 20 min. He had tertiary level education. Formal review of brain and chest imaging performed as part of his cancer staging demonstrated calcification of the falx, tentorium and ligaments, and tentorium cerebelli. No vertebral or rib anomalies were detected. Review of his orthopantomography (OPG) identified a Stafne cyst but no odontogenic keratocysts. Ophthalmologic assessment identified bilateral increased pigmentation of the choroid and retina. Proband 1 met GS clinical diagnostic criteria, fulfilling two major criteria (> 5 BCCs in lifetime and falx calcification) and two minor criteria (macrocephaly and ocular anomalies, including hypertelorism and pigmentary changes of the retinal epithelium).

Proband 1 underwent extensive germline and somatic investigations via both clinical diagnostics and research pathways (Table 2). Initial germline NGS panel testing (*BAP1*, *CDK4*, *CDKN2A*, *FH*, *FLCN*, *POT1*, *PTCH1*, *RB1*, *SUFU*) identified no reportable variants, including PV/LPV or variants of uncertain significance (VUS). Extended testing of *PTCH2* was also uninformative. He was consequently enrolled in two genomic research studies: the Genetic Cancer Risk in the Young (RisC) study and the Cancer Molecular Screening and Therapeutics (MoST) trial (Additional file 1). In RisC, germline whole-genome sequencing (WGS) of peripheral blood DNA (average depth 35×) detected no PV/LPV, covering known cancer susceptibility genes, including *PTCH1*. Concurrent somatic testing via the MoST study (TST170 platform) of a metastatic axillary lymph node revealed four PV: NM*_*000264.3(*PTCH1)*:c.1228_1229del p.(Ser410Cysfs*26) (VAF 39.7%), NM_000546.5(*TP53*):c.994–2 A > G (VAF 37.9%), biallelic *CDKN2A* loss, and NM_198253(*TERT)*:c.-124 C > T promoter variant (VAF 32.8%). Subsequent clinical tumor sequencing (Tempus xT panel) confirmed the same variants. Variant curation of *PTCH1* c.1228_1229del is summarized in Table 2. To evaluate the possibility of a cryptic *PTCH1* germline variant impacting splicing, RNA analysis of *PTCH1* was performed on peripheral blood samples. No splice variants were detected. To investigate mosaicism, DNA from cultured skin fibroblasts collected from normal-appearing, but sun-exposed skin above the left elbow and non–sun-exposed skin from the left buttock was sequenced, revealing the *PTCH1* variant at 2.62% and 1.14% VAF, respectively. Manual reanalysis of peripheral blood DNA identified the variant at 1% VAF, confirming low-level mosaicism (Additional file 2).

**Table 2:** Summary of genetic investigations and *PTCH1* variant curation and allele frequency for presented probands

	Proband 1	Proband 2	Proband 3	Proband 4
***PTCH1*** ** variant**	NM_000264.3(*PTCH1*):c.1228_1229del *p*.(Ser410Cysfs*26)	NM_000264.5(*PTCH1*):c.258_259del *p*.(Leu87Ilefs*2)	NM_00264.3(*PTCH1*):c.1093 C > T *p*.(Gln365Ter)	NM_000264.3 (*PTCH1*):c.3169–2 A > G *p*.(Val1057_Leu1102del)
**Variant classification**	Pathogenic	Pathogenic	Pathogenic	Likely Pathogenic
**Evidence for pathogenicity**	This two base pair deletion in exon 9 results in a frameshift and introduces a premature stop codon, leading to a truncated PTCH1 protein with predicted loss-of-function. This variant has not been reported in ClinVar or LOVD databases but is recognized in the COSMIC database as a confirmed somatic variant in medulloblastoma. Loss-of-function variants in *PTCH1* are known to be pathogenic [20, 27]	Located in exon 2, the variant is predicted to cause a reading frameshift at codon 87 and introduction of a premature stop codon two amino acids downstream. This variant is absent from population databases and has been reported in at least three GS affected patients in the literature (PMID 8981943, 16301862). Other diagnostic laboratories have reported the variant as pathogenic in ClinVar (Variation ID: 219697).	Located in exon 8, this variant was predicted to result in a premature stop codon, leading to a truncated PTCH1 protein with predicted loss of PTCH1 protein function via nonsense mediated decay. This variant is recorded several times in ClinVar as pathogenic and has been reported in at least one individual with GS (PMID: 29575684; 31374299). Other truncating variants in this exon, for example c.1120G > T p.(Glu374Ter), c.1138G > T p.(Glu380Ter) and c.1148 C > A p.(Ser383Ter), are recorded in ClinVar as pathogenic. This variant has not been observed in population database gnomAD v2 and v3 non-cancer.	This single nucleotide substitution at the − 2 position in intron 18 is predicted by in silico splicing prediction programs (MaxEnt, NNSPLICE and SSF) to result in complete loss of the canonical acceptor spice site and possible exon 19 skipping p.(Val1057_Leu1102del) with predicted nonsense mediated decay. Published RNA studies show that this variant (also referred to as NM_000264.3 (*PTCH1)*:c.3157–2 A > G) results in whole exon skipping, leading to an in-frame 46-amino acid deletion, which abrogates the transmembrane domains 9 and 10 of the PATCHED protein [34]. This variant has not been reported in ClinVar but is recorded in the LOVD database as pathogenic and was previously reported in an individual with GS [34]. A different variant at this canonical splice site, c.3169-1G > A, is recorded in ClinVar as likely pathogenic. This variant has not been observed in population database (gnomAD).
**Summary of germline investigations**				
*PTCH1* gene sequencing (peripheral blood DNA)		No reportable variants detected in *PTCH1* (aged 29)^a^		
NGS Panel (peripheral blood DNA)	Panel: *BAP1*,* CDK4*,* CDKN2A*,* FH*,* FLCN*,* POT1*,* PTCH1*,* RB1*, and *SUFU*^*a*^ No reportable variants detected (aged 38)	Panel: *PTCH1*,* PTCH2* and *SUFU*^*a*^ No reportable variants detected (aged 33)	Panel: *PTCH1* and *SUFU*^*a*^ *PTCH1*:c.1093 C > T detected at 11% VAF. No reported variants detected in *SUFU* (aged 32)	Panel: *BAP1*,* CDK4*,* CDKN2A*,* FH*,* FLCN*,* POT1*,* PTCH1*,* RB1* and *SUFU*^*a*^ *PTCH1*:c.3169–2 A > G detected at 15% VAF. No reported variants detected in the other genes listed (aged 30)
Updated testing (peripheral blood DNA)	No reportable variants in *PTCH2*^*b*^ (aged 38)	Panel: *PTCH1* and *SUFU*^*a*^ No reportable variants were detected with a target read depth of 100x and VAF of approximately 20% (aged 39).		
Research testing (peripheral blood DNA)	WGS with targeted panel analysis^c^ No reportable variants detected (aged 39)			
RNA studies (peripheral blood)	No variants impacting splicing were detected^d^			
Manual reanalysis of peripheral blood DNA	Same *PTCH1* variant detected at ~ 1.0% VAF^h^	Same *PTCH1* variant detected in 4 reads^h^		
**Summary of somatic investigations**				
Tumor DNA (1)	Metastatic lymph node (Left axilla)^e^ *PTCH1* variant detected at 39.7% VAF	BCC - left dorsal second toe: *PTCH1* and *SUFU*^f^ Same *PTCH1* variant detected at 14.5% VAF		
Tumor DNA (2)	Repeat biopsy of Metastatic lymph node (Left axilla)^g^ Same *PTCH1* variant detected at 58.4% VAF	BCC - right deltoid: *PTCH1* and *SUFU*^f^ Same *PTCH1* variant detected at 18.1% VAF		
Tumor DNA (3)		BCC - left upper calf: *PTCH1* and *SUFU*^f^ Same *PTCH1* variant detected at 2.2% VAF		
Skin fibroblast DNA (sun exposed)	Above left elbow: Same *PTCH1* variant detected at 2.62% VAF^h^			
Skin fibroblast DNA (non-sun exposed)	Left buttock: Same *PTCH1* variant detected at 1.14% VAF^h^			

Abbreviations: BCC, basal cell carcinoma; NGS, Next-generation sequencing; VAF, variant allele frequency; WGS, whole-genome sequencing

^a^ Performed by the Peter MacCallum Cancer Centre Molecular Genetics Laboratory

^b^ Performed by invitae

^c^ Performed as part of the Genetic Cancer Risk in the Young (RisC) study. The RisC study investigated the heritable aspects of cancer in a young population suggestive of genetic etiology. See Additional file 1 for full list of genes analysed

^d^ Performed by Genomic Medicine laboratory, Saint Mary’s Hospital in Manchester (UK)

^e^ Performed as part of the Molecular Screening and Therapeutics (MoST) study. The MoST trial aimed to identify targetable somatic variants in rare and poor prognosis cancers [35]. See Additional file 1 for full list of genes analysed

^f^ Performed by SA Pathology, Adelaide SA, Australia

^g^ 596-gene Tempus xT Assay (Tempus Labs Inc., Chicago, USA) [36]. Proband 1 elected to self-fund additional treatment-focused somatic testing after disease progression, and this was arranged by his medical oncologist. Note: Tempus used different RefSeq - reported as *PTCH1* (NM_001083602): c.1030_1031del p.(Ser344fs)

^h^ Skin fibroblast DNA and peripheral blood DNA further analysed by Peter MacCallum Cancer Centre Molecular Genetics using the Low Frequency Variant Detection tool in QIAGEN’s CLC Genomics Workbench (v12.0.2), supported by sequencing data with read depths > 500x as their germline variant caller was not validated to detected mosaic/low VAF variants

Proband 1’s further systemic treatment included Hedgehog inhibitor (HHI) (sonidegib), immunotherapy (durvalumab and tremelimumab), chemotherapy (carboplatin and paclitaxel) and other trial treatments. However, overall, he had progressive disease with brain and bone metastases and was treated with palliative radiotherapy. He died 19 months following initial presentation at age 40.

Following his death, predictive testing in his sons (IV-1 and IV-2; Fig. 1a) confirmed absence of the *PTCH1* variant. Neither of the children met GS clinical criteria. Follow-up is planned at age 20 or earlier for review. In the interim, sun-protection was advised given the family history and skin type.

## Case presentation 2

Proband 2 (III-1 in Fig. 1b) presented at age 39 years for updated genetics assessment in the context of a long-standing clinical diagnosis of GS. Her review was prompted by her son’s dermatologist to guide his ongoing need for clinical surveillance. Her first BCC was diagnosed at the age of 19 years, and she had since had over 250 BCCs removed (histologically confirmed). The lesions have a predilection for the right side of her body. While a couple of BCCs were previously removed from her groin, the remainder were in sun exposed areas. Given the burden of lesions, at the time of review, she was undergoing regular general anesthesia every three months for batched lesion removal with her plastic surgeon. She had a right odontogenic keratocyst removed at age 30 years following diagnosis at 27 years. She subsequently had two further suspected right-sided keratocysts diagnosed at age 39 years after imaging for an overlying post-surgical skin infection. Findings on OPG were of two well defined radiolucent lesions within the right mandibular ramus measuring 4 mm and 7 mm in size with well corticated margins. She reported being advised she had an extra rib during pregnancy; however, the imaging was not available for review. On examination, she had macrocephaly (OFC 57.8 cm, > 99th percentile, + 3.1 SD) with no dysmorphic facial features. She had bilateral multiple palmar pits and multiple widespread skin lesions suspicious for BCCs. Falx calcification was incidentally identified at age 39. She recently had surgery for suspected bilateral ovarian fibromas; though the final pathology revealed features suggestive of ovarian follicular cysts.

Proband 2 met the clinical diagnostic criteria, fulfilling four major criteria (> 5 BCCs in lifetime and first lesion < 30 years, falx calcification, odontogenic keratocysts and multiple palmar pits) and at least one minor criteria (macrocephaly). The additional minor criterion of an extra rib was not counted given the diagnostic imaging was unable to be reviewed. Confirmation of the rib anomaly was not arranged as further criteria were not required for a clinical diagnosis and to avoid unnecessary radiation exposure.

Proband 2 had previously undergone *PTCH1* sequencing followed by peripheral blood NGS panel targeting *PTCH1*,* PTCH2* and *SUFU* at ages 29 and 33, respectively with no reportable variants detected. On review, updated germline testing was performed, sequencing coding regions and flanking splice sites of *PTCH1* and *SUFU* (mean coverage 100×; ~20% VAF threshold). Again, no reportable variants were identified.

**Fig. 2:**
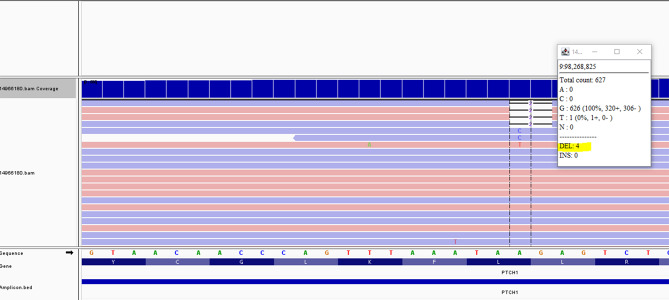
Integrative genomics viewer image of Proband 2’s peripheral blood analysis detecting the same 2 bp deletion in *PTCH1* in only four reads

Somatic testing was subsequently performed on three BCCs excised from distinct anatomical sites: left dorsal second toe, right deltoid region, and left upper calf. Deep sequencing of *PTCH1* and *SUFU* (mean coverage 677×, 871×, and 781×, respectively) identified the same NM_000264.5(*PTCH1*):c.258_259del p.(Leu87Ilefs*2) variant in all three lesions (VAFs of 14.5%, 18.1%, and 2.2%, respectively). Variant curation is summarized in Table 2. Manual reanalysis of peripheral blood sequencing data revealed the variant at a VAF < < 1% (4 reads), consistent with low-level mosaicism (Fig. 2).

She was counseled regarding the post-zygotic origin of her *PTCH1* variant and that except for her children, no other relatives are considered at risk of the variant. Predictive testing was offered to her sons to guide ongoing surveillance, with testing confirming neither had inherited the *PTCH1* variant. She plans to commence targeted systemic therapy with a HHI in the coming months.

## Case presentation 3

Proband 3 (III-2 in Fig. 1c) was referred by her dermatologist at age 32 years due to a long-standing history of BCCs, with over 50 lesions excised since adolescence. She reported no history of severe sunburn and consistent sun protection. At age 24 years, OPG revealed radiolucent lesions in the mandible and maxilla, with histology confirming odontogenic keratocysts. Examination showed macrocephaly (OFC 58.5 cm, > 99th percentile, + 3.8 SD) and 2–3 palmar pits bilaterally. Prior MRI brain incidentally identified falx calcification. Proband 3 met GS clinical diagnostic criteria, fulfilling four major criteria (> 5 BCCs in lifetime, falx calcification, odontogenic keratocysts and palmar pits) and one minor criterion (macrocephaly). Additional minor criteria, such as vertebral/rib and ocular anomalies, were not formally assessed.

Targeted sequencing of *PTCH1* and *SUFU* performed on peripheral blood DNA identified a NM_00264.3(*PTCH1)*:c.1093 C > T p.(Gln365Ter) variant at 11% VAF (mean coverage ~ 700×) (Additional file 2). Variant curation is summarized in Table 2. The low VAF was consistent with a diagnosis of mosaic GS. Further tissue testing was not pursued as the molecular diagnosis was established.

Proband 3 commenced treatment with sonidegib under the care of her dermatologist, which has so far continued for 11 months. Existing lesions have remained stable and no new BCCs have developed. Proband 3 was counseled that her future children may be at risk, with recurrence risk up to 50%, and reproductive options were discussed. Testing of other relatives was not indicated.

## Case presentation 4

Proband 4 was a 30-year-old male (III-1 in Fig. 1d) clinically diagnosed with GS at age 15 years, based on multiple odontogenic keratocysts, palmar and plantar pits during childhood, and young-onset BCC. He sought updated genetic evaluation to inform reproductive planning. On most recent assessment, he had a height of 185 cm (89th percentile), OFC 59 cm (> 99th percentile + 2.7 SD), and bilateral palmar pits, accentuated with warm water immersion. Falx calcification was noted prior to age 30; though the exact age is unclear. Since age 15, he has required excision of 3–4 BCCs annually, with increased lesion burden during his twenties. Over a recent 4-month period, he underwent 15 BCC excisions. He practices sun protection, and his current treatment includes Niacinamide.

Genetic testing was undertaken to confirm a likely clinical diagnosis of GS, based on the presence of four major criteria. A 9-gene NGS panel (*BAP1*,* CDK4*,* CDKN2A*,* FH*,* FLCN*,* POT1*,* PTCH1*,* RB1 and* SUFU) was requested. A NM_000264.3(*PTCH1)*:c.3169–2 A > G p.(Val1057_Leu1102del) intronic splice-site variant was detected at approximately 15% VAF, suggestive of mosaicism (Additional file 2). Variant curation is summarized in Table 2. Subsequent predictive testing confirmed that neither parent (II-9 and II-10 in Fig. 1d) carried this variant, supporting a *de novo* mosaic origin.

Following his genetics review, Proband 4 has proceeded to have a son (IV-1 in Fig. 1d) who does not presently meet clinical GS criteria and is not macrocephalic. Predictive testing for the *PTCH1* LPV will be pursued via a paediatric genetics service guided by shared decision making between the clinician and family.

## Literature review

A search of the *Pubmed* and *Google Scholar* databases for terms including GS, GGS, NBCCS, mosaicism, segmental, *PTCH1*,* PTCH2*, and *SUFU* was performed. Full text articles that were written in English were included, with selection based on review of title and abstract, or full text review when the abstract was inconclusive for a mosaic presentation. Some articles were also identified by backward reference searching, if identified as a citation of one of the other referenced articles in this manuscript. Articles discussing skin lesions only, such as basal cell naevi, basaloid follicular hamartoma, comedones, and basal cell epitheliomas, that did not explicitly address clinical assessment for GS or report a molecular diagnosis were excluded. We identified a total of 15 mosaic probands (11 previously published cases and our four probands). Clinical presentation and molecular results are summarized in Table 1. Additionally, articles that discussed possible GS mosaicism, including suspected gonadal mosaicism, but where the clinical features of GS were not available were excluded from the review although are listed in the footnote of Table 1. Additionally, two reports of somatic *SMO* variants (included in the footnote of Table 1) were also excluded from the review, as subsequent publications have countered these probands have an overlapping condition, rather than GS [37, 38].

Review of the literature demonstrates considerable variability in the presentation of mosaic *PTCH1* and/or clinically diagnosed GS probands, with some fulfilling the diagnostic criteria and others with only minimal features. A limitation of this comparison is that the authors likely used different thresholds to define a proband as having mosaic GS or to report the clinical impact of *PTCH1* mosaicism. The most common clinical manifestations included multiple BCCs (*n* = 14, 93% of probands), palmar/plantar pits (*n* = 7, 47%), calcification of the falx (*n* = 7, 47%) and odontogenic keratocysts (*n* = 7, 47%). Skeletal and ocular abnormalities were observed in only four (27%) and two (13%) probands, respectively. None of the reported mosaic probands presented with syn/polydactyly, lympho-mesenteric or pleural cysts, ovarian or cardiac fibromas or cleft lip and/or palate. Though it must be acknowledged that ascertainment bias is likely to impact clinical recognition of the syndrome in this context. It is also likely that probands were not assessed for all possible manifestations of GS, which would also limit the utility of this evaluation.

For comparison, Huq et al. [39] reported phenotypic features in a cohort of 19 Australian individuals with GS. This cohort was a mix of patients with molecularly confirmed *PTCH1* or *SUFU* PV and clinically diagnosed or highly suspected cases (mosaicism was not excluded). The predominant features in their cohort were BCCs (77.8%), macrocephaly (66.7%), odontogenic keratocysts (63.2%), plantar/palmar pits (55.6%) and falx calcification (64.3%). Overall, the proportion of patients in their cohort with BCCs and palmar/plantar pits were similar to our reviewed mosaic cases, with a lower proportion of odontogenic keratocysts and falx calcification in the confirmed mosaic probands. Of note, all four of our presented probands had confirmed (*n* = 3) or reported (*n* = 1) macrocephaly, though only one additional reviewed mosaic proband was reported to have macrocephaly. Interestingly, the number of GS features reported in mosaic probands does not appear to correlate with the mutation burden, with one mosaic individual presenting with only multiple BCCs despite having a *PTCH1* PV at 23–74% VAF in non-cancer tissues [17] and others having multiple clinical features, despite very low levels (~ 1%) in peripheral blood DNA [13]. This is most striking in Proband 2 who had a significant BCC burden but a detected *PTCH1* PV of < < 1% VAF in peripheral blood DNA.

## Discussion

Here, we report four cases of GS resulting from mosaic *PTCH1* variants, as well as a comprehensive literature review of mosaic presentations. Extensive germline evaluation employing clinical NGS panels and/or research WGS did not identify a causative variant for Probands 1 and 2. Whilst *PTCH2* has been disputed as a GS gene [3] this was evaluated in these two probands for completeness. Somatic tumor sequencing was critical in establishing a molecular diagnosis in both cases, with retrospective peripheral blood analysis revealing the *PTCH1* variant at surprisingly low VAFs. In contrast, mosaicism was more readily identified in Probands 3 and 4, with blood-based VAFs of 11% and 15%, respectively, despite their classic phenotypes. These cases expand our understanding of the phenotypic variability and molecular spectrum associated with *PTCH1* mosaicism and highlight the importance of evaluating multiple tissues when a genetic etiology cannot be detected on routine germline testing.

Somatic mosaicism refers to the occurrence of two (or more) genetically distinct cell populations within an individual derived from post-zygotic mutation(s) [33]. In contrast to inherited variants, somatic mosaic variants may only affect a portion of the body and can only be transmitted to offspring if the variant is present in gametes [40]. In cancer genetics, it is important to distinguish tumour-restricted somatic variants from mosaic or germline variants that are detectable in other tissues, because only the latter carry broader clinical and hereditary implications. Indeed, over 85% of sporadic BCCs harbor somatic *PTCH1* variants [41]. In one study, 64% of patients diagnosed with BCC before age 40 years had somatic *PTCH1* variants, but only one patient had two BCCs with the same variant, raising suspicion for GS mosaicism, although clinical confirmation was not possible [25]. As demonstrated in Proband 2, detection of the same *PTCH1* PV in two or more independent BCCs provides strong evidence for postzygotic mosaicism [42]. In Proband 1, who met clinical GS criteria, detection of a *PTCH1* variant in one BCC raised the possibility of mosaicism. Although archival tissue from earlier tumors was unavailable, the same variant was subsequently identified at low VAF (< 3%) across multiple non-neoplastic tissues. The pathogenicity of the variant was not in question, with frameshift variants causing premature termination of the resulting protein being the most common mechanism in GS [43]. Additionally, the *PTCH1* c.1228_1229del variant has only been reported once in COSMIC (COSM255305), unlike more commonly recurring variants such as c.3944 C > T (*n* = 52), c.3606del (*n* = 41), and c.3921del (*n* = 28), making recurrent independent somatic events unlikely and supporting a mosaic diagnosis.

According to the classification proposed by Martinez-Glez et al. [40], Proband 1 would be considered to have “mild involvement” (F1), with < 10% of tissues affected and < 5% VAF. Notably, despite this low-level mosaicism, he exhibited a classic clinical phenotype, with a high BCC burden and multiple additional diagnostic features. This discordance between variant burden and phenotypic expression highlights the complexity of mosaic GS and suggests that even low-level postzygotic variants may lead to clinically significant disease depending on developmental timing and tissue distribution. Supporting this, BCC burden has been shown not to differ between individuals with confirmed germline *PTCH1* PVs and those with suspected mosaicism meeting clinical criteria [7].

Recent clinical overlap between segmental GS, Curry-Jones syndrome and Happle-Tinschert syndrome, caused by somatic *SMO* variants has been debated, with the terms ‘Mosaic Hedgehog Spectrum’ and somatic *SMO* syndrome now proposed [37, 38, 44–46]. As included in the footnote of Table 1, segmental GS attributed to somatic *SMO* mosaicism was reported in two cases though no germline (constitutional) *SMO* PV have been reported [29, 30]. Proband 1 had no reportable somatic variants in *SMO*, as determined by MoST and Tempus testing. This was not assessed for our other probands. To the best of our knowledge, there are no reported cases of *SUFU* or *PTCH2* mosaicism in the literature conferring a *classic* GS phenotype. However, van Dal et al. [47] reported a patient with mosaic *SUFU* PV (VAF 8% in non-lesional skin and 15% in peripheral blood) with *Multiple Hereditary Infundibulocystic Basal Cell Carcinoma syndrome*. This may be considered a reduced penetrance phenotype, but the authors propose individuals with *SUFU* PV should be considered a distinct group [47]. This proband was a 72-year-old female who had developed 20 BCC from age 57 years and had plantar (but no palmar) pitting. This patients’ phenotype matched that of two other probands with heterozygous *SUFU* PV, reinforcing our hypothesis that mosaicism in the Hedgehog signaling pathway can still present as a classic phenotype. Ongoing delineation of the clinical phenotypes caused by PV within this spectrum will aid consistency in formal diagnosis and could be important to optimize patient management.

Somatic mosaicism may be challenging to detect or assess, especially when present at only low levels in typically sampled DNA sources, such as peripheral blood and skin [33]. Three probands in the reviewed studies had PVs that were entirely absent from peripheral blood. In one of these probands the PV was also absent from non-lesional skin, and in another the PV was present in only 0–9% of non-lesional skin. Like Probands 1 and 2, Reinders et al. [13] report one proband with four GS features (three major and one minor) with a *PTCH1* PV detected at only ~ 1% in peripheral blood DNA. It is important to highlight potential confounders in our interpretation of Proband 1’s data. Firstly, analysis of DNA from cultured skin fibroblasts may not indicate the true VAF in our proband. Selective advantage for the fibroblast cells *without* the PV in the fibroblast culturing microenvironment is possible and could result in higher VAF for the wild type allele. Moreover, as both skin biopsies were taken from the same side of the proband’s body, a segmental effect cannot be excluded, whereby the areas of biopsied skin happened to have a lower frequency of the *PTCH1* PV. Indeed, 40% of the case reports reviewed in Table 1 demonstrate segmental mosaicism.

Mosaicism typically arises *de novo* during embryogenesis. Mosaicism can also arise due to ‘genetic rescue’ of an inherited variant, where a proportion of cells revert to the wild-type (or an alternate) sequence. This phenomenon has been reported in conditions ranging from immunodeficiency (e.g. Wiskott–Aldrich syndrome), skin disorders (e.g. epidermolysis bullosa) and cancer predisposition (e.g. Bloom syndrome and Fanconi anemia) [48, 49], including GS [23]. The lack of a clinical diagnosis in our probands’ parents, argues against a ‘genetic rescue’ event in our presented cases and supports *de novo* somatic mosaicism.

Another possible explanation is a different cryptic germline *PTCH1* PV undetectable with routine clinical testing technology (e.g. deep intronic variants). Bholah et al. [31] reported this as a mechanism in GS. These cryptic variants may be detectable with RNA studies. Evaluation of RNA in Proband 1 did not identify any cryptic variants impacting splicing. 

Although BCCs rarely metastasize, this has been reported in several GS patients [50–53], including Proband 1. Oral and more recently, topical HHIs have proven useful for treatment of BCCs, albeit complicated by side-effects and therapeutic resistance [54]. However, objective response rates to HHIs in metastatic disease are low (8–49%) compared to 46–71% in locally advanced disease [55]. Like Proband 1, *PTCH1* and *TP53* co-mutations have been reported in 16% of young-onset BCCs [25] and 42% of sporadic tumors [16]. Neither of these studies evaluated an impact on prognosis, though Jayaraman et al. [56] hypothesized the high mutational burden of BCCs may induce a greater immune response and result in less aggressive disease. There may be other modifying genetic factors that could account for the difference in therapy response to what would be expected for GS. Undeniably, Proband 1’s response to HHI was limited in both extent and duration, even though the *PTCH1* PV was detected at ~ 40–58% VAF in tumor tissue. Co-occurring *TP53* mutation may have contributed to therapeutic resistance. These findings underscore the need to understand how somatic progression and mutational burden affect treatment response in mosaic GS.

## Genetic counseling implications

There is well-established evidence of clinically diagnosed [8, 9] and molecularly confirmed mosaic presentations of GS [12, 13, 14] and these cases demonstrate the importance of considering postzygotic *PTCH1* mosaicism as a mechanism that may explain unresolved cases. We propose that the presented case series supports Reinders et al.’s [12] assertion that even low-level *PTCH1* mosaicism can confer a strong phenotype for GS. Nonetheless, two of our probands were referred to genetics approximately 15 years after the first feature of GS manifested, demonstrating a need for improved syndrome recognition. Admittedly, several features of GS are not typically apparent at a young age [57] and the genetics referral criteria relies on accumulation of phenotypic features to be cost-effective. However, failure to identify children and adolescents or young adults with GS may result in a missed opportunity for early sun prevention behaviors and appropriate clinical interventions. With predominant referrals from maxillofacial surgeons and dermatologists [39], targeted education of these professionals may assist in earlier GS recognition. 

The presented cases demonstrate the need for careful clinical assessment and genetic investigation to differentiate autosomal dominant from *de novo* and/or mosaic disease. This is critically important in Australia given the overall higher incidence of skin cancers due to environmental factors, leading to potential phenocopies and assumptions of autosomal dominant inheritance. In clinically suspicious cases, it is important to consider somatic sequencing of two (or more) BCCs or if tumor tissue is unavailable normal skin fibroblasts (with intentional biopsy of normal skin bilaterally to minimize possible segmental effects) along with high read depth or alternate sequencing methodologies (e.g. MLPA and digital-droplet PCR) [17] to thoroughly assess for mosaicism.

It also reiterates the importance of assessing for mosaicism following uninformative germline testing in those with a clinical diagnosis of GS to inform genetic counseling for at-risk relatives. Once a molecular diagnosis is established, predictive genetic testing for relatives becomes possible. As illustrated in our case series, multiple factors may influence the timing of predictive testing, including clinician discretion, parental anxieties or family preference. Proband 1’s family pursued predictive testing to clarify risk for his children, which provided timely reassurance following his death. In the other cases, confirmation of mosaicism enabled informed reproductive decision-making and planning for future offspring, including predictive testing timed to clinical need. Importantly, the recurrence risk to offspring of mosaic probands may be less than the 50% risk typical for heterozygous carriers [40]. Accordingly, as demonstrated in Proband 4, a mosaic molecular diagnosis may also increase reproductive confidence. We recommend all offspring be referred to genetics for clinical examination and discussion of the merits of pursuing predictive testing, using a shared decision-making approach. Confirmation of a child’s genetic status prior to achieving a diagnosis based on their clinical features, will enable age-appropriate surveillance, including screening for cardiac fibromas and odontogenic keratocysts in early childhood and initiation of sun-safe behaviors. As approximately 25% of female GS patients will develop ovarian fibromas, with a high frequency of bilateral lesions, fertility preservation is also an important consideration when planning management or surgical interventions [58]. Moreover, a direct benefit of establishing a molecular diagnosis is to allow consideration of pre-implantation genetic testing, an assisted reproduction technique, in which only embryos lacking the causative variant are selected for implantation [59]. Taken together, the clinical management of subsequent generations can be appropriately supported through confirmation of a molecular diagnosis. Genetic counseling should incorporate discussion of mosaicism as a mechanism of disease, including limitations of conventional testing, and the utility of tumor and multi-tissue sequencing where suspicion remains high.

## Conclusion

This Australian case series and international literature review provides a comprehensive evaluation of mosaic GS phenotypes and is intended as a resource to improve identification of mosaic probands. It highlights the complexities in achieving a molecular diagnosis, and that the degree of mosaicism does not always correlate with disease severity. Importantly, it also justifies why evaluation for mosaicism is needed in unexplained cases, with a molecular diagnosis benefiting both the individuals’ clinical management and informing at-risk relatives. Earlier detection of GS may promote sun protective behaviors to potentially minimize BCC development or facilitate prompt intervention with targeted therapies leading to better cosmetic and clinical outcomes.

## Supplementary Information

Below is the link to the electronic supplementary material.

Supplementary Material 1: Additional File 1: Gene set from the RisC and MoST studies (pdf)

Supplementary Material 2: Additional File 2: Integrative genomics viewer images of Proband 1, 3 and 4’s germline and somatic testing (pdf)

## Data Availability

No datasets were generated or analysed during the current study.

## Abbreviations

%: Percentile
BCC: Basal cell carcinoma
BCN: Basal cell naevi
ca: Cancer
CNV: Copy number variant
Dx: Diagnosed
F: Female
GS: Gorlin syndrome
GGS: Gorlin-Goltz syndrome
HHI: Hedgehog inhibitor
LOH: Loss of heterozygosity
LPV: Likely pathogenic variant
M: Male
MLPA: Multiplex ligation-dependent probe amplification
mo: Months old
MoST: Molecular Screening and Therapeutics
MRI: Magnetic resonance imaging
NBCCS: Nevoid Basal Cell Carcinoma syndrome
NGS: Next-generation sequencing
NLS: Non-lesional skin
OFC: Occipitofrontal circumference
OPG: Orthopantomography
PB: Peripheral blood DNA
PV: Pathogenic variant
RisC: Genetic Caner Risk in the Young study
SCC: Squamous cell carcinoma
UDEC: Urine derived endothelial cells
VAF: Variant allele frequency
WGS: Whole-genome sequencing; yo: years old
