# Supplementary material for: Challenging interpretation of low-level PTCH1 mosaicism in patients with clinically diagnosed Gorlin syndrome: a case series and review of the literature
Source: Hered Cancer Clin Pract. 2026 Mar 26;24:10. doi: 10.1186/s13053-026-00332-3 (PMC13141527; doi:10.1186/s13053-026-00332-3)
Supplement: Supplementary file 1 — Supplementary Material 1: Additional File 1: Gene set from the RisC and MoST studies (pdf) [file 13053_2026_332_MOESM1_ESM.pdf]

Additional file 1:

**RISC gene set:**

Cancer: *APC, BMPR1A, BRCA1, BRCA2, CDC73, CDH1, CDKN2A, DICER1, FH, FLCN, MUTYH, MEN1, MLH1, MSH2, MSH6, NF1, NF2, PALB2, PTCH1, PMS2, POLD1, POLE, PTEN, RET, SDHAF2, SDHB, SDHC, SDHD, SMAD4, STK11, SUFU, RB1, TP53, TSC1, TSC2, VHL, WT1*

Non-cancer: *ACTA2, ACTC1, APOB, ATP7B, CACNA1S, COL3A1, DSC2, DSG2, DSP, FBN1, GLA, KCNH2, KCNQ1, LDLR, LMNA, MYBPC3, MYH7, MYH11, MYL2, MYL3, OTC, PCSK9, PKP2, PRKAG2, RYR1, RYR2, SCN5A, SMAD3, TGFBR1, TGFBR2, TMEM43, TNNI3, TNNT2, TPM1*

**MoST gene set**:

Methods: Targeted sequence analysis was performed at Garvan Institute of Medical Research. DNA and RNA was extracted from FFPE tumor material using Qiagen AllPrep DNA/RNA FFPE kit, and libraries were created and enriched using the Illumina TruSight Tumor 170 reagents kit (Illumina product OP-101-1003) including DNA and RNA probes panel. This panel enriches for coding regions and splice sites from 110 genes for DNA, and 60 genes for RNA. Samples were uniquely indexed, pooled and sequenced on an Illumina NextSeq500 to generate 2x100bp reads at a target coverage of 500 reads/base. Somatic analysis was performed by identifying rare (population allele frequency < 0.1%) genetic variants within targeted exons + essential splice site regions, detected in tumor tissue by VarDict, which identifies small insertions, deletions, and single nucleotide variants (SNVs). Somatic copy number analysis was performed using CNVkit to identify copy number alterations. Copy number estimates were adjusted for estimated tumor purity. RNA fusion analysis was conducted using both STARFusion and FusionCatcher. Scope of test: This test considered somatic point mutations, short insertions and deletions, and somatic copy number alterations.

**DNA**

*AKT1 BTK CSF1R FBXW7 FGFR4 KMT2A MUTYH PIK3CB RICTOR AKT2 CARD11 CTNNB1 FGF1 FLT1 KRAS MYC PIK3CD ROS1 AKT3 CCND1 DDR2 FGF10 FLT3 LAMP1 MYCL1 PIK3CG RPS6KB1 ALK CCND2 DNMT3A FGF14 FOXL2 MAP2K1 MYCN PIK3R1 SLX4 APC CCND3 EGFR FGF19 GEN1 MAP2K2 MYD88 PMS2 SMAD4 AR CCNE1 EP300 FGF2 GNA11 MCL1 NBN PPP2R2A SMARCB1 ARID1A CD79A ERBB2 FGF23 GNAQ MDM2 NF1 PTCH1 SMO ATM CD79B ERBB3 FGF3 GNAS MDM4 NOTCH1 PTEN SRC ATR CDH1 ERBB4 FGF4 HNF1A MET NOTCH2 PTPN11 STK11 BAP1 CDK12 ERCC1 FGF5 HRAS MLH1 NOTCH3 RAD51 TERT BARD1 CDK4 ERCC2 FGF6 IDH1 MLLT3 NPM1 RAD51B TET2 BCL2 CDK6 ERG FGF7 IDH2 MPL NRAS RAD51C TFRC BCL6 CDKN2A ESR1 FGF8 INPP4B MRE11A NRG1 RAD51D TP53 BRAF CEBPA EZH2 FGF9 JAK2 MSH2 PALB2 RAD54L TSC1 BRCA1 CHEK1 FAM175A FGFR1 JAK3 MSH3 PDGFRA RAF1 TSC2 BRCA2 CHEK2 FANCI FGFR2 KDR MSH6 PDGFRB RB1 VHL BRIP1 CREBBP FANCL FGFR3 KIT MTOR PIK3CA RET XRCC2*

**RNA**

*ABL1 BRCA1 ERG FGFR1 JAK2 MSH2 NTRK2 PPARG AKT3 BRCA2 ESR1 FGFR2 KDR MYC NTRK3 RAF1 ALK CDK4 ETS1 FGFR3 KIF5B NOTCH1 PAX3 RET AR CSF1R ETV1 FGFR4 KIT NOTCH2 PAX7 ROS1 AXL EGFR ETV4 FLI1 KMT2A NOTCH3 PDGFRA RPS6KB1 BCL2 EML4 ETV5 FLT1 MET NRG1 PDGFRB TMPRSS2 BRAF ERBB2 EWSR1 FLT3 MLLT3 NTRK1 PIK3CA*
