# Supplementary material for: Challenging interpretation of low-level PTCH1 mosaicism in patients with clinically diagnosed Gorlin syndrome: a case series and review of the literature
Source: Hered Cancer Clin Pract. 2026 Mar 26;24:10. doi: 10.1186/s13053-026-00332-3 (PMC13141527; doi:10.1186/s13053-026-00332-3)

Additional file 2

Proband 1

Sample Details: Testing was performed on DNA extracted from fresh tissue (cultured skin fibroblasts), received from NSW Health Pathology. This variant was detected at approximately 1.14% variant allele frequency (VAF) in DNA extracted from cultured skin fibroblasts of the patient.

IGV: Proband 1 Skin biopsy (non-sun exposed)

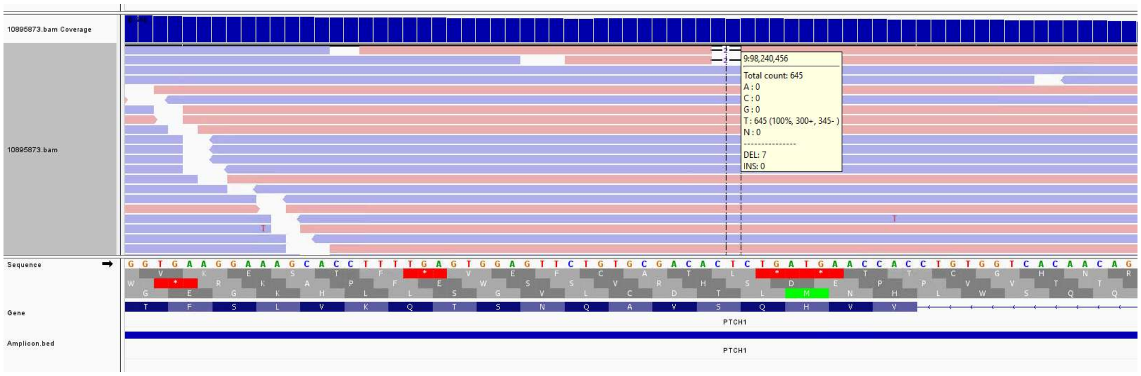

IGV: Proband 1 Skin biopsy normal tissue (sun exposed)

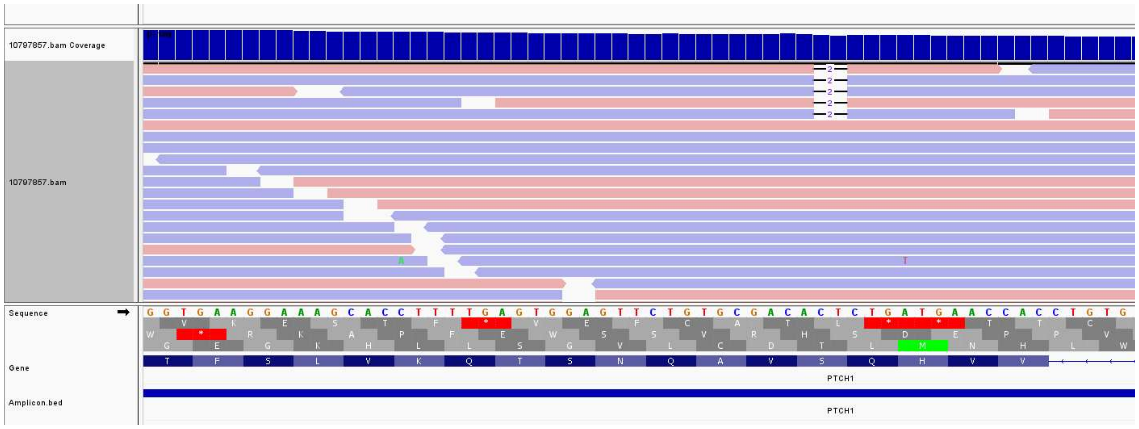

IGV: Proband 1 Skin biopsy (sun exposed)

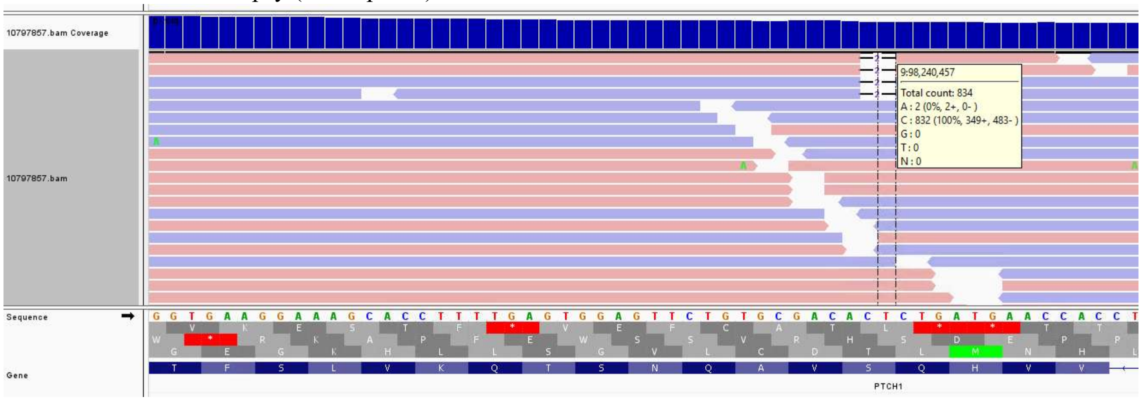

Proband 3:

Sample details: Peripheral blood DNA

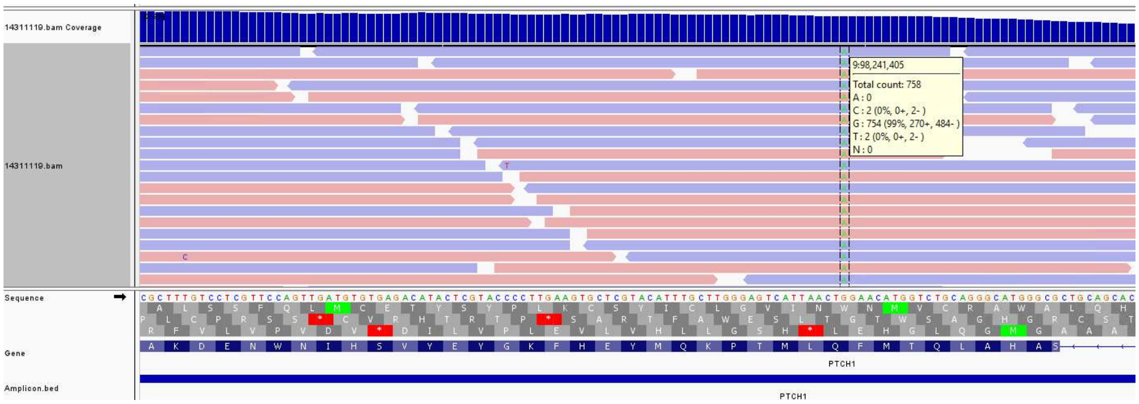

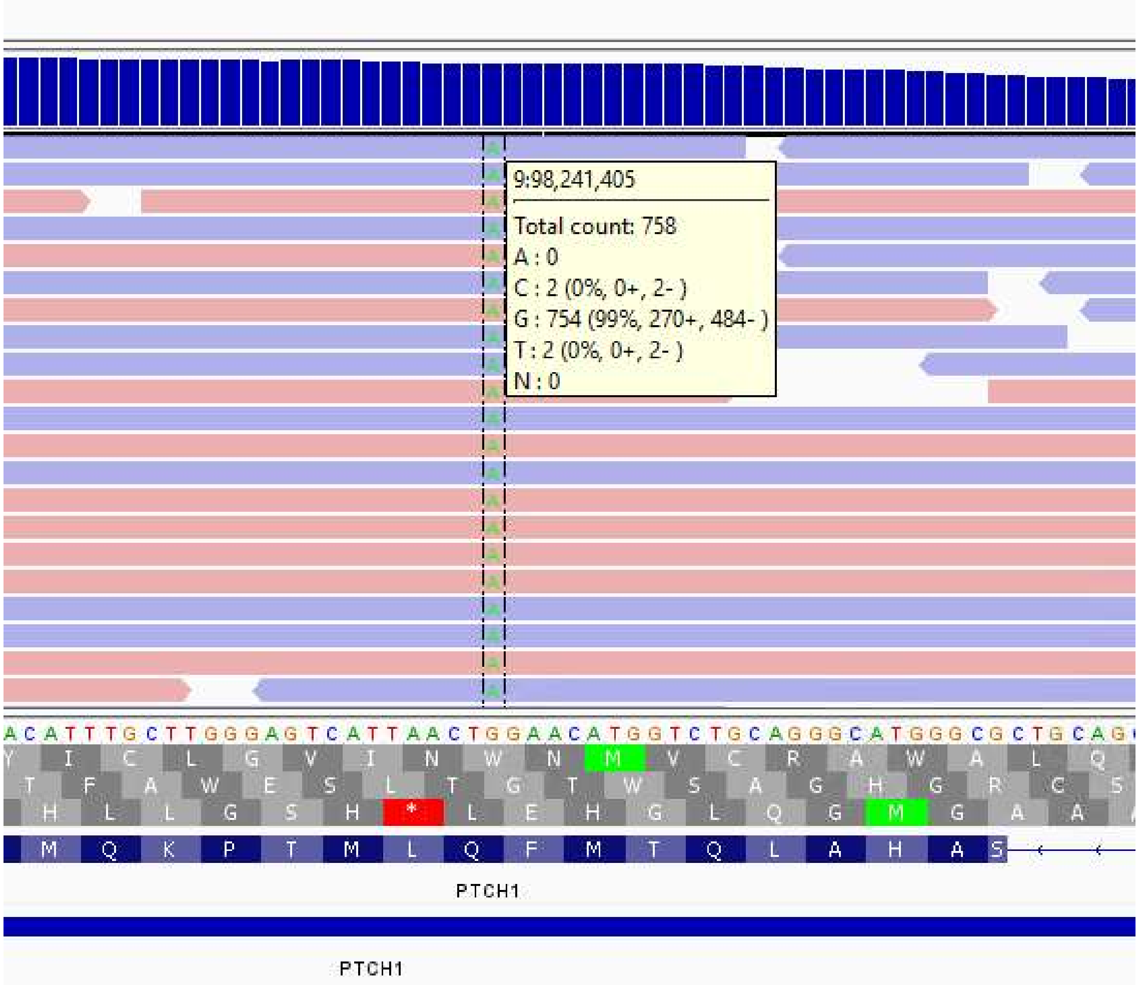

Proband 4

Sample details: Peripheral blood DNA

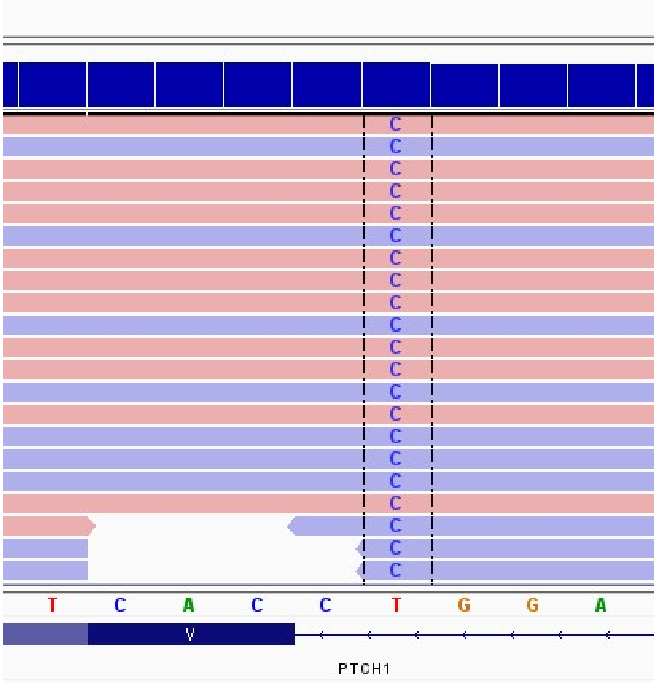

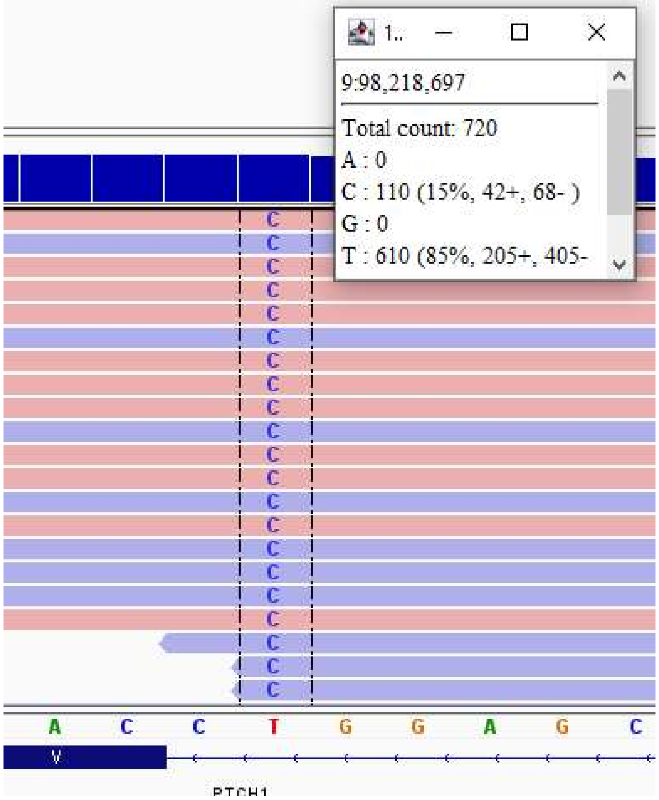

Supplement: Supplementary file 2 — Supplementary Material 2: Additional File 2: Integrative genomics viewer images of Proband 1, 3 and 4’s germline and somatic testing (pdf) [file 13053_2026_332_MOESM2_ESM.pdf]
